# Comparison of the Utility of Recombinant B8/2 Subunit of the Antigen B, Native Antigen, and a Commercial ELISA Kit in the Diagnosis of Human Cystic Echinococcosis

**DOI:** 10.29252/.23.4.246

**Published:** 2019-07

**Authors:** Amir Savardashtaki, Zohreh Mostafavi-Pour, Farzaneh Arianfar, Bahador Sarkari

**Affiliations:** 1 Department of Medical Biotechnology, School of Advanced Medical Sciences and Technologies, Shiraz University of Medical Sciences, Shiraz, Iran; 2 Recombinant Proteins Laboratory, Department of Biochemistry, School of Medicine, Shiraz University of Medical Sciences, Shiraz, Iran; 3 Department of Parasitology and Mycology, School of Medicine, Shiraz University of Medical Sciences, Shiraz, Iran

**Keywords:** Diagnosis, *Echinococcus granulosus*, Hydatid cyst

## Abstract

**Background:**

Cystic echinococcosis (CE) is a helminthic disease caused by the larval form of *Echinococcus granulosus*. In the present study, the B8/2 subunit of antigen B (AgB) of *E. granulosus* was expressed in *E. coli* host and then applied in a diagnostic ELISA set up.

**Methods:**

The DNA sequence of AgB8/2 subunit from *E. granulosus* was extracted from the GenBank and codon-optimized according to *E. coli* codon usage. The target sequence was cloned in an expression vector (pGEX-4T-1). The produced antigen was used in an ELISA system, and its performance for the diagnosis of human hydatid cyst was evaluated, using sera from CE and non-CE patients, along with the sera from healthy subjects. Moreover, the diagnostic value of the recombinant protein was compared with native AgB, as well as with a commercial kit.

**Results:**

Antibodies to hydatid cyst were detected in 27 out of 30 patients corresponding to a sensitivity of 90% (95% CI: 73-98%). Cross-reaction with sera of non-CE subjects was seen in two cases resulted in a specificity of 93.5% (95% CI: 82-98%) for the test. A sensitivity of 87% and specificity of 90% were found for the native form of the antigen, while the ELISA commercial kit had a sensitivity of 97% and specificity of 95%.

**Conclusion:**

Our data show that rEgAgB8/2 is an appropriate source of antigen for the serological diagnosis of human hydatid cyst. Co-expression of the rEgAgB/2 along with other subunits of AgB may enhance the performances of these antigens for the serodiagnosis of human CE.

## INTRODUCTION

Cystic echinococcosis (CE), or hydatid cyst, is a chronic parasitic disease that is caused by the larval form or metacestode of *Echinococcus granulosus*[1]. The cyst is mainly localized in human liver and lung, and its clinical presentation may range from an asymptomatic infection to a severe and fatal disease [1,2]. CE is considered as an important economic and public health concern in several developing countries including Iran [1,3]. This disease affects about 1.2 million people worldwide, and the global burden of the disease is estimated to be 188,000 new cases per year[1,3]. In Iran, the annual number of CE surgeries has been reported to be 1295 cases, accounting for a mean annual surgical incidence of 1.6 in 100,000 inhabitants[4,5]. Seroepidemiological surveys have indicated asymptomatic CE in 7.3% of the individuals in the rural areas of Iran’s southeastern province of Kerman and 5.6% in the volunteer blood donors from Fars Province in southern Iran[6,7]. CE diagnosis is often confirmed by a combination of imaging techniques along with serological approaches. So far, several serological methods, based on antigen or antibody detection, have been utilized for the diagnosis and follow-up of CE with satisfactory results, but far from being optimal due to their suboptimal sensitivities or specificities[1,8-12].

The development of DNA recombinant technology has facilitated the generation of recombinant peptides suitable for highly sensitive approaches in the diagnosis of different infectious diseases including CE[13-16]. Several antigens of *E. granulosus* have been evaluated in different serodiagnosis assays of human hydatid cyst[8,13]. Among them, antigen 5 (Ag5) and antigen B (AgB) are frequently used antigens. AgB, known as the main protein responsible for metabolic adaptation and survival of the parasite, is the prominent antigen of hydatid cyst fluid. The antigen is a lipoprotein of 120-160 kDa, consisted of a group of about 8-kDa subunits. Under reducing conditions in SDS-PAGE, it dissociates into 8, 16, and 24 kDa subunits[17,18]. The 8-kDa subunit, the smallest one, has presented the best performance in the diagnosis of CE and has been extensively used in synthetic, recombinant, or native forms in different serological assays[10,12,14,19-21].

Molecular studies have demonstrated that AgB is encoded by a multigene family that is variably expressed, with at least five major gene clusters named EgAgB1-5[18]. The putative protein isoforms encoded by the five EgAgB genes differ 44–81% in amino acid sequence[18]. A comparison between the AgB8/1 and AgB8/2 nucleotide sequences has showed 53.5% identity among exons and 50% identity between introns of these two subunits[22]. Accordingly, the immunodiagnostic performances of different subunits of AgB (EgAgB8/1-5) seem to be different. Formerly, it has been demonstrated that the recombinant EgAgB8/2 (rEgAgB8/2) has a higher diagnostic value in comparison with the rEgAgB8/1 or native AgB for the serodiagnosis of human CE[22].

In our previous study, we successfully cloned and expressed the EgAgB8/1 subunit of AgB and evaluated its diagnostic efficacy in CE. A sensitivity and specificity of 93% and 92% was found for the EgAgB8/1 subunit[14]. In the current study, we focused on other subunits of the antigen where the EgAgB8/2 was expressed in *E. coli* host, and its performance for serological diagnosis of human CE was evaluated using an ELISA system.

## MATERIALS AND METHODS

### Construction, optimization, and cloning of AgB8/2

Construction, optimization, and cloning of rEgAgB8/2 were performed as previously described with some modifications[14]. Briefly, the DNA sequence of AgB8/2 was extracted from NCBI GenBank database with the accession number of DQ835667.1. The target sequence was optimized based on *E. coli* codon usage and the optimized gene fragment was synthesized (Biomatik, Ontario, Canada) and cloned in pBluescript II SK (+) vector. The fragment was subsequently excised by *Eco*RI/*Xho*I digestion and subcloned in pGEX-4T-1 (GE Healthcare, Illinois, US) expression vector and transformed into *E. coli* DH5α. The recombinant plasmid was extracted using QIAprep Spin Miniprep Kit (Qiagen, Hilden, Germany). The extracted plasmid was transformed into BL21 (DE3)pLysS cells for expression under the T7 promoter. The expression of AgB8/2 was induced by 0.5 mM IPTG (Invitrogen, USA) at 37 °C for four hours.

### Protein purification

The cells were harvested by centrifugation (1800 ×g at 4 °C for 10 min), and the resultant pellet was re-suspended in a lysis buffer (Triton-X100, PBS [pH 8], and 0.5 M EDTA) and sonicated (5 × 30 s) on ice. The resultant cell lysate was centrifuged at 15,000 ×g at 4 °C for 30 min and incubated at -20° C overnight. After that, the clear supernatant was collected, and the recombinant protein was purified by affinity chromatography, based on its glutathione S-transferase (GST)-tag, using immunoaffinity column (Sigma-Aldrich, Germany). The bound protein was eluted with elution buffer (reduced glutathione, 50 mM Tris, pH 8). Finally, desalination of purified protein was done using a dialysis membrane in PBS (pH 7.5)[14].

### SDS-PAGE and Western blotting

SDS-PAGE of the recombinant and the native protein (native AgB purified from hydatid cyst fluids as described previously)[21] were carried out on 18% (w/v) polyacrylamide gel containing 0.1% SDS as documented before[14].

### Patients and control sera

Hydatid cyst patients’ sera were obtained from 30 pathologically-confirmed CE patients. In addition, 32 samples of non-CE patients, including subjects affected by giardiasis (n = 3), hymenolepiasis (n = 3), toxocariasis (n = 3), toxoplasmosis (n = 3), fascioliasis (n = 3), malaria (n = 2), cryptosporidiosis (n = 2), trichostrongyliasis (n = 1), patients with autoimmune diseases (n = 9; including three with vitiligo, three with Behçet’s disease, and three with systemic lupus erythematous) as well as patients with FUO (fever of unknown origin; n =3) were collected. Moreover, 30 sera were collected from the healthy subjects who had no history of CE and were all seronegative when tested for anti-hydatid cyst antibodies, using AgB-ELISA[10]. The study was approved by the Ethical Committee of Shiraz University of Medical Sciences (Ethical code number: IR.SUMS.REC. 1393.S7376), and an inform consent was obtained from those who voluntarily donated the samples or from their patients.

### ELISA using native and recombinant antigen

ELISA with native AgB and rEgAgB8/2 was performed as previously described[14]. Briefly, 96-well microplates were sensitized with 5 μg/mL of either rEgAgB8/2 or native AgB. Unbound sites were blocked with non-fat skimmed milk. Sera samples of CE patients along with positive and negative controls were added to the microplates. Conjugated goat anti-human IgG was used to detect the antigen-antibody complex and a substrate (o-phenylenediamine; 0.4 mg/mL) was used to visualize the reaction. The cut-off point was considered as 2SD above the mean of control samples.

### ELISA with the commercial kit

All the sera samples were analyzed by a commercial ELISA kit (Euroimmune, Germany), available for the diagnosis of CE, based on the manufacturer’s guidelines.

### Statistical analysis

The diagnostic accuracy of the native AgB, rEgAgB/2, and the commercial kit was verified by the area under the receiver operating characteristic curve, using SPSS software (ver. 20). Cut-off values were established for the test based on Youden’s Index. Cohen’s kappa statistic was used to find out the extent of agreement between ELISA systems, using different antigens, and also the commercial kit.

## RESULTS

### Production and purification of rEgAgB8/2

**Fig. 1:**
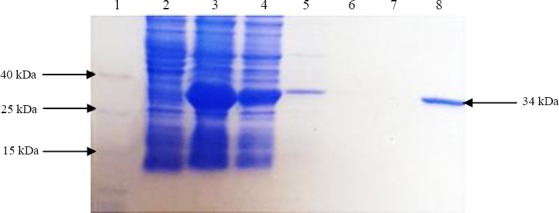
SDS-PAGE analysis of the rEgAgB8/2 protein expression and purification. Lane 1, protein weight marker; lanes 2 and 3, uninduced and induced cultures of *E. coli* by IPTG; lane 4, prewash; lanes 5, 6, and 7, consecutive washes; lane 8, final elution (GST-AgB8/2).

**Fig. 2:**
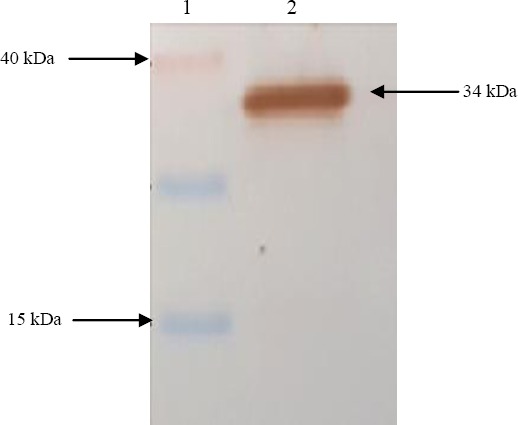
Western blotting analysis of rEgAgB8/2 expression in *E. coli*. Lane 1, molecular weight marker; Lane 2, rEGAgB8/2 fused with GST tag.

After cloning a part of the AgB along with the signal sequence (rEgAgB8/2) in a pGEX-4T-1 vector, the correct cloning procedure was confirmed through the sequencing of the expression unit. The expression of the rAgB8/2 with the estimated molecular weight of 34 kDa was confirmed by SDS-PAGE analysis (Fig. 1). Reactivity of the recombinant protein with sera of CE patients was further confirmed with Western blotting (Fig. 2). A GST agarose resin was used for the purification of the developed recombinant protein.

### Diagnosis of human CE using rEgAgB8/2

GST-containing rEgAgB8/2 was used in the ELISA system to check its performance for the detection of the anti-hydatid cyst antibodies. Using an ELISA checkerboard, the optimum concentrations of the rEgAgB8/2, horseradish peroxidase-conjugated IgG, and the serum were determined as 5 µg/mL, 1:100, and 1:4000 dilutions, respectively. Using the developed ELISA system, anti-hydatid cyst antibodies were detected in the sera of 27 out of 30 CE patients corresponding to a sensitivity of 90% (95% CI: 73-98%). The test had a specificity of 93.5% (95% CI: 82-98%) since cross-reaction with non-CE cases was seen in 2 out of 30 samples. None of the healthy subjects were positive by the developed ELISA system. The performance of the ELISA, using the rEgAgB8/2, the ELISA commercial kit, and native AgB for the diagnosis of human hydatid cyst are shown in Table 1. Native AgB was able to detect anti-hydatid cyst antibodies in the sera of 26 out of 30 (86.7%) of CE cases. Six cases of controls were also positive in AgB-ELISA. Accordingly, a sensitivity of 87% and specificity of 90% were calculated for the native AgB. A sensitivity of 97% and specificity of 95% were also found for the ELISA commercial kit. The performances of the rEgAgB8/2, native antigen, and the commercial kit for the diagnosis of CE are shown in Table 2. There was a good agreement among the three assays. The kappa coefficient (*k*) for rEgAgB8/2 and native AgB was 0.786, for native AgB and the commercial kit was 0.76, and for rEgAgB8/2 and the commercial kit was 0.83.

**Table 1:** The performance of rEgAgB8/2, native antigen B, and commercial kit for the diagnosis of CE

Type of serum	Number	Positive cases in commercial kit No. (%)	Positive cases in native-AgB ELISA No. (%)	Positive cases in rEgAgB8/2 ELISA No. (%)
CE patients	30	29 (97)	26 (87)	27 (90)
Vitiligo	3	0	0	0
Behçet’s disease	3	0	0	0
Lupus	3	0	0	0
Giardiasis	3	0	1 (33)	0
Hymenolepiasis	3	0	0	0
Toxocariasis	3	1 (33)	0	0
Toxoplasmosis	3	0	1 (33)	1 (33)
Fascioliasis	3	0	1 (33)	1 (33)
FUO	3	0	1 (33)	0
Malaria	2	1 (50)	0	0
Cryptosporidiosis	2	0	0	0
Trichostrongyliasis	1	0	0	0
Healthy subjects	30	1 (3.3)	2 (6.7)	2 (6.7)

**Table 2:** Relative performances of the rEgAgB8/2, the native antigen, and the ELISA commercial kit for the diagnosis of CE

Type of antigens	Sensitivity (%) (95% CI)	Specificity (%) (95% CI)	PPV (%) (95% CI)	NPV (%) (95% CI)
rEgAgB8/2	90 (73-98)	93.5 (82-98)	88 (67-95)	95 (86-99)
Native AgB	87 (68-95)	90 (79-96)	81(63-92)	93(83-98)
Commercial kit	97 (81-100)	95 (85-99)	90 (74-97)	98 (90-100)

PPV, positive predictive value; NPV, negative predictive value

## DISCUSSION

The primary asymptomatic CE may last for years and the late diagnosis may be associated with inadequate care, resulting in the disease spread and poor clinical outcomes. In contrast, timely diagnosis of the CE offers better opportunities for timely intervention and control of the disease. Diagnosis of CE is based on imaging modalities along with serological approaches[8]. Serological assays along with the imaging approaches would confirm the diagnosis of CE. Low sensitivity and also low specificity render the optimal performance of such approaches for proper diagnosis of the disease.

To overcome the problem of low sensitivity and cross-reactivity, utilizing the recombinant antigens in the diagnosis of hydatid cyst has gained much attention[23,24]. In the previous study, we produced a recombinant antigen, AgB8/1, as a primary target for the development of an immunoassay for the diagnosis CE with satisfactory results[14]. In the current study, production of EgAgB8/2, as a specific and highly abundant antigen of *E. granulosus* was considered. The five subunits of AgB have relatively same molecular weights, but the amino acid sequences of these subunits differ as much as 44–81%. Thus, their performances in the immunodiagnosis of hydatid cyst seem to be different. Using several approaches, a high level of the heterologous expression of the EgAgB8/2 fragment was achieved. We effectively take advantage of codon optimization to get a high-level expression of EgAgB8/2 as a eukaryotic antigen in *E. coli* as a prokaryotic cell factory.

In our pilot study, at first, AgB8/2 was used without codon optimization but proper expression level of the desired protein was not achieved. As it has been shown in previous studies[25-27] the codon optimization increases the heterologous expression of the recombinant proteins in *E. Coli*. Hence, we decided to optimize AgB8/2 DNA for bacterial expression, using Genscript’s OptimumGene™ design platform, which employs a unique algorithm and proprietary codon usage table. Besides, codon adaptation index was one of the parameters analyzed in our study. When this index is more than 0.8, a high expression level of protein can be expected. For our protein, this index was 0.4 before codon optimization and increased to 0.9 after that. The other significant factor is codon preference. The most frequent codons worth 100, and when the frequency of these codons increases in one sequence, the chance of expression increases as well. This value was 44% for AgB8/2 before codon optimization and after that was improved up to 75%. Based on above optimization, a final concentration of 4 mg/mL of the purified EgAgB8/2 protein was obtained, which is higher than the yield of AgB8/1 in our previous study[14].

The antigen was expressed as a GST-tagged fusion protein to enable GST affinity purification, enhance protein expression and increase the solubility of the produced protein. It has been shown that the n-terminus fusion of the target recombinant protein with one of the host proteins will increase the yield of expression as well as its solubility and reduces the development of inclusion bodies[28-30]. Considering this point, in the present study, the protocol for native purification was followed to keep the native construction of the protein and also to prevent denaturing of the protein and to keep the intact conformational epitopes. Enzymatic cleavage, by thrombin, can simply be used to remove the tag. No differences in the immunoreactivity of the antigens were seen when the rEgAgB8/2, with or without tag, was evaluated for the detection of anti-hydatid cyst antibodies. Therefore, we assumed that fusion tag did not interfere in the antigen-antibody reaction in our ELISA system. In order to be in a safe side and to avoid the antigen degradation or possible alteration in the conformational epitopes of the antigen by enzymatic reactions, which are necessary for the cleavage of the tag, we left the antigen coupled with the tag. Furthermore, immunoreactivity analysis revealed that GST tag usually does not affect the performance of the tests since they do not cross-react with antibodies present in the human serum.

The sensitivity and specificity of the produced EgAgB8/2 antigen in this study were 90% and 93.5%, respectively, which were higher than those of the native counterpart. It appears that pairing this antigen with EgAgB8/1 antigen may improve the performance of the test for the diagnosis of human hydatid cyst. Mohammadzadeh *et al*.[12] have achieved similar results with recombinant antigen from Japanese isolates of *E. granulosus*. In a study in China, Jiang *et al*.[30] have indicated that the sensitivity of the EgAgB8/1 antigen is greater than that of EgAgB8/2 in the diagnosis of human CE. Comparison of the performances of EgAgB8/1 produced in our previous study with EgAgB8/2 subunits revealed that the sensitivity of AgB8/1 antigen is greater than AgB8/2. This result is consistent with Jiang *et al*.^’^s[30] study in which they reported a higher sensitivity for AgB8/1 in comparison with AgB/2[30].

Pairwise comparison of the immunoreactivity results of the sera with AgB8/1 and AgB8/2 demonstrated that some samples are reactive with one of these antigens but not with the other one. This situation may reflect the different status of the cyst in the host and the dominance of the expression of one of AgB subunits. Recently, immunoproteomics studies has demonstrated a difference in the expression of immunogenic antigens of hydatid cysts in the CE1 and CE2 stages where it was found that in the CE1 stage, the AgB8/1 expression level was higher, while in the CE2 stage, the expression level of AgB8/2 was greater[31,32]. This claim may describe why some sera samples are reactive with AgB8/1 but not with AgB8/2 antigen[22].

Findings of the current study reveal that gene codon optimization and fusion with GST tag sequence facilitate both the heterologous expression of the rAgB8/2 antigen in *E. coli* and its purification. The purified recombinant protein is a good antigenic source for the serological diagnosis of CE. Co-expression of the EgAgB/2 along with EgAgB/1 and application of these two proteins in an ELISA system may improve the performances of these recombinant antigens in the diagnosis of CE. Further studies are needed to clarify the diagnostic value of other subunits of AgB, particularly EgAgB/4, utilizing a native strain of *E. granulosus*.
